# Depth Model and 5-Axis Variable-Angle Laser Engraving Experiment Based on the Energy Conservation Principle

**DOI:** 10.3390/mi13122228

**Published:** 2022-12-15

**Authors:** Pengpeng Sun, Qiang Liu, Jian Wang, Liuquan Wang, Zhenshuo Yin

**Affiliations:** 1 School of Mechanical Engineering and Automation, Beihang University, Beijing 100191, China; 2 Research and Application Center of Advanced CNC Machining Technology and Innovation, Beijing 100191, China; 3 Jiangxi Research Institute, Beihang University, Nanchang 330096, China; 4 National Center for Science & Technology Evaluation, Beijing 100081, China

**Keywords:** energy conservation principle, 5-axis variable-angle laser engraving, depth model, adaptive control

## Abstract

To ensure the consistency of laser engraving depth in chemical milling, the precise control of 5-axis variable-angle laser engraving was the focus of research. Based on the energy conservation principle, the depth model of 5-axis variable-angle laser engraving is established, and the relationships among the laser engraving depth, laser power, scanning velocity, and beam axis angle are proposed. A depth-constraint real-time adaptive control method of laser power is proposed considering the variable scanning velocity and beam axis angles. The depth model parameters are identified by an orthogonal experiment, and a variable-angle laser engraving experiment with adaptive control of laser power is carried out. The coefficient of determination of the proposed depth model is 0.977, which means that the engraving depth model established in this paper predicts the engraving depth effectively and reliably. The depth-constraint adaptive control method of laser power obtains stable and uniform machining results under abrupt changes in scanning velocity and beam axis angles.

## 1. Introduction

**Figure 1:**
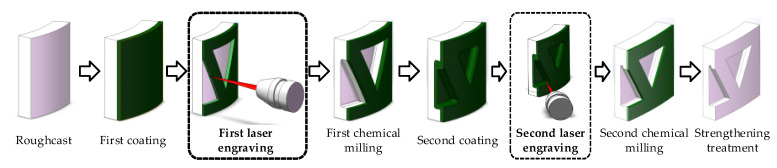
Chemical milling processing diagram of an aero-engine casing body.

In order to meet the light weight requirements for aerospace structural parts, a large number of thin-walled parts with complex features have been designed and widely applied, which are usually processed by chemical milling for material removal after mechanical milling [1,2]. As the pre-process for chemical milling, engraving processing is the process of cutting the geometric pattern of the protective adhesive and peeling it off according to the area which requires chemical milling without damaging the basis material [3]. Laser engraving is the most common method; using the laser to ablate the protective adhesive and remove the protective layer on the metal surface. In order to achieve maximum weight reduction, the thin-wall parts often need to be engraved and chemical-milled twice, as shown in Figure 1. The second laser engraving must be processed on the side wall of the cavity formed by the first chemical milling. The laser beam axis is usually tilted towards the sidewall surface because of the interference of the laser head. At the corner, the angles of the beam axis relative to the sidewall surface change dramatically, and the laser scanning velocity significantly decreases due to the dynamic constraints of the machining equipment. Using fixed laser parameters will lead to inconsistency in the engraving depth at different positions and severe over burning at the corner. Precise control of engraving depth under variable beam axis angle and scanning velocity is a critical problem of the second laser engraving, which has essential engineering significance for high-efficiency and high-quality processing [4].

Scholars mainly study and select laser engraving parameters by experiment. Gnanamuthu, D.S. [5] and Slysh, P. [6] applied different lasers to etch the protective adhesive layer with a given thickness on planar parts, and verified the feasibility of the laser engraving method. Griffin, B.M. [7] and Leone, C. [8] explored the influence of laser power and feed rate parameters on engraving quality and engraving depth. Gao, X.J. et al. [9,10,11,12] studied the influence of laser power, engraving velocity, laser frequency, and incident angle on the engraving depth through a single-factor experiment and determined the trend through the linear regression method. In order to realize the precise control of laser ablation for complex geometric feature patterns, theoretical models of laser etching have been studied. The existing methods of research into engraving depth models are mainly divided into three categories, including the laser ablation mechanism method, the regression analysis method, and the artificial intelligence method, which are shown in Table 1.

In summary, the existing studies on engraving parameters, theoretical models, and control methods mainly focus on selecting process parameters and the processing control of simple geometric feature patterns. An advantage of the depth model based on the laser ablation mechanism is that it has a complete theoretical basis, but a disadvantage is that it has a large correlation with the material, so it needs a targeted overview of the relevant model to identify the parameters of the model. The engraving depth model based on regression analysis can accurately analyze the relationship between single laser process parameters and engraving depth. Meanwhile, multiple data regression is limited by scholars’ model selection and understanding, and different models may present different analysis results. The engraving depth prediction model based on the artificial intelligence method can realize the results of the fast prediction model but lose information about the physical processes and cannot accurately predict the influence of various laser parameters on the marking depth. Although the results can provide a reference for laser processing of complex geometric feature patterns, it is challenging to meet the requirements of the second laser engraving process with variable beam axis angles in terms of accuracy and effectiveness. 

In this article, a depth model of 5-axis variable-angle laser engraving is established based on the energy conservation principle and realizes real-time adaptive adjustment of laser power with the change of scanning velocity and beam axis angle under the constraint of target depth to obtain a stable engraving process and uniform depth results. The rest of this article is organized as follows: Section 2 introduces the experimental platform, the engraving orthogonal experiment, and laser power adjustment method. The engraving depth model is elaborated in Section 3. Then, the experiment results and discussion are detailed in Section 4. Finally, Section 5 concludes this article.

## 2. Materials and Methods

### 2.1. Experimental Equipment

**Figure 2:**
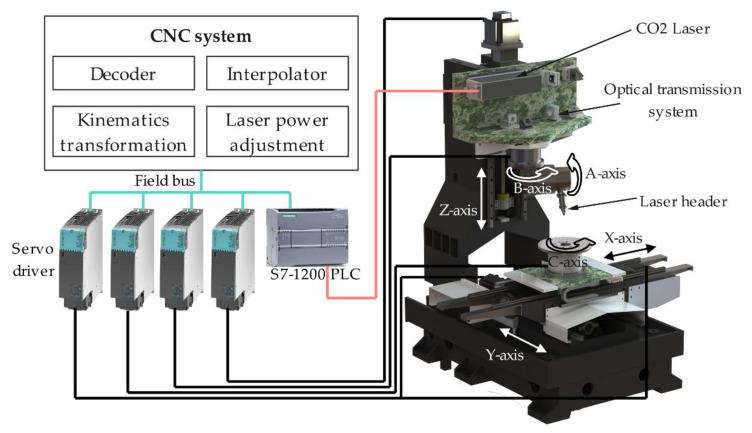
The architecture of the 6-axis 5-linkage laser engraving experimental platform.

The experimental equipment used in this work is a 6-axis 5-linkage laser engraving platform with 3 linear axes and 3 rotary axes, as shown in Figure 2. The rotary table C-axis installed on the XY cross slide performs circumferential indexing and positioning of the work piece. The double swing head consisting of A and B axes is installed on the Z-axis carriage. A CO2 quasi-continuous laser was selected as the laser source and fixed to a marble on the Z-axis. A space-flexible optical transmission system was designed to achieve the accurate spatial direction of the laser beam through a series of mirrors and a pair of orthogonal hollow torque motors. Part of the laser parameters in the machining area are shown in Table 2. The laser scanning movement of variable-angle laser engraving is realized by the linkage motion of the X, Y, Z, A, and B axes through the RTCP (Rotated Tool Center Point) function.

The CNC system consists of a decoder, interpolator, kinematics transformation, laser power adjustment, and other modules. The NC command text is decoded by the decoder and interpolated in real-time. The kinematics transformation obtains the position commands in the machine tool coordinate system based on the motion chain structure. The commands are sent to the servo drivers to control the movement of feed axes. The output power and frequency of the CO_2_ laser are controlled by the input PWM signal, which is converted in real-time following the laser parameter command using the pulse generator module of Siemens SIMATIC S7-1200 PLC.

The relationship between the average output power of the laser and the PWM signal duty cycle was detected by a laser power meter (Type: Ophir L50(150)A-BB-35), as shown in Table 3.

### 2.2. Orthogonal Test Design of Laser Engraving

**Figure 3:**
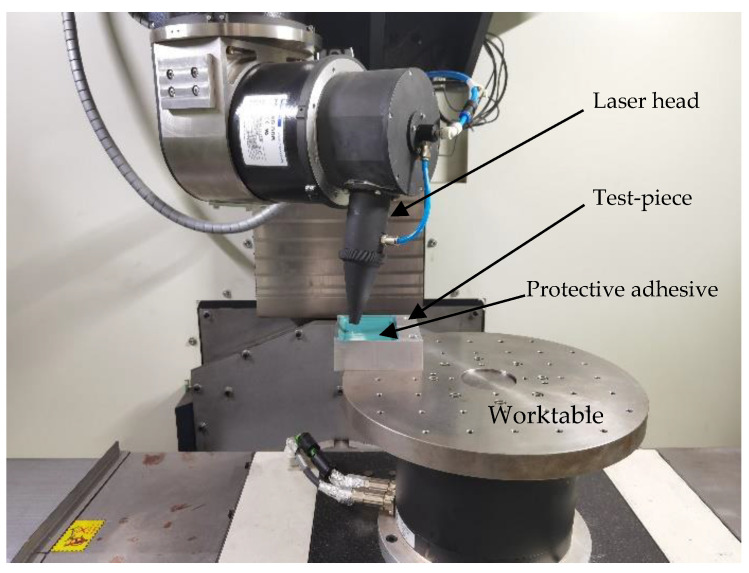
The process of the laser engraving orthogonal test.

The specification of the test piece with aluminum alloy 7075 as the substrate material was 100 mm × 100 mm. The piece surface was covered with AC850 chemical milling protective adhesive with a thickness of about 1mm. An orthogonal test of laser engraving was designed to provide representative data for the parameter identification of the depth model. Table 4 shows the test parameters. The tests were carried out on the experimental platform, as shown in Figure 3.

The 3D (three-dimensional) profiles of the processing results were measured by an ultra-depth microscope (Type: Olympus DSX1000) with an objective lens, DSX10-XLOB20X. The measured field of view was 953 μm × 953 μm, and the magnification was 320 times.

### 2.3. Laser Power Adjustment of the 5-Axis Variable-Angle Laser Engraving Process

**Figure 4:**
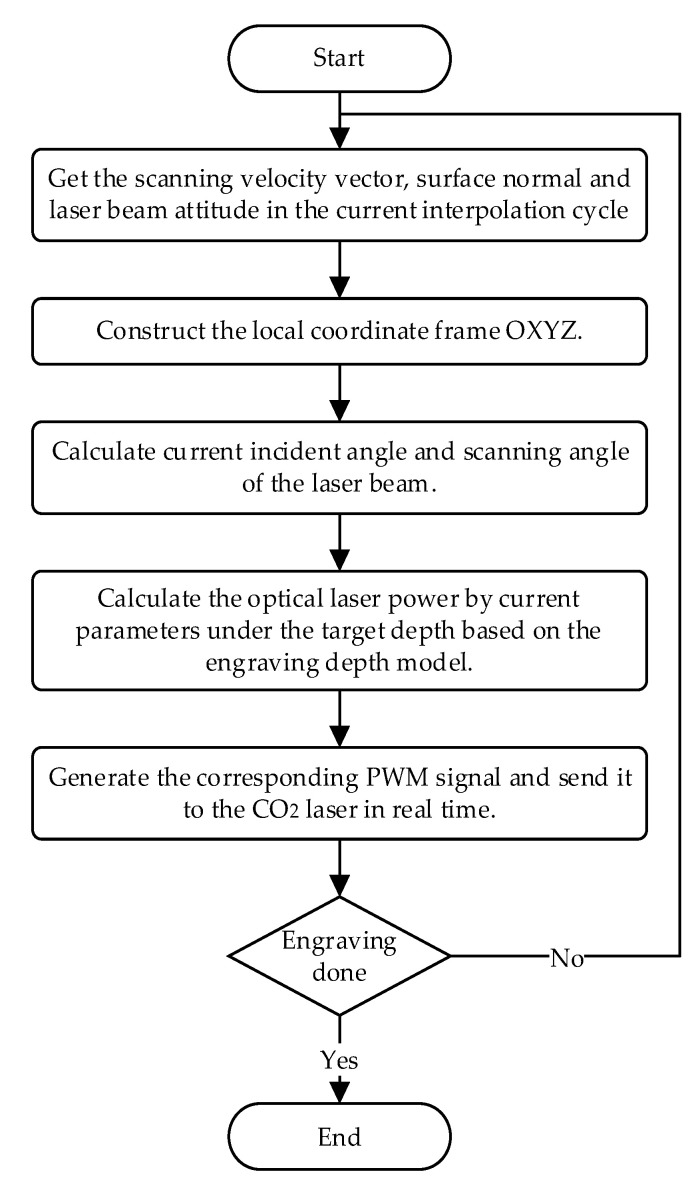
Flow chart of real-time laser power adjustment in the engraving process.

In the process of 5-axis variable-angle laser engraving, the engraving trajectory and the adhesive surface determine the laser incident angle and scanning angle. The CNC system controls the laser power and scanning velocity. Therefore, the proposed depth model can adjust laser output power in real time according to the target depth and current scanning velocity during processing to achieve uniform engraving depth. A laser power adaptive adjustment method is put forward to maintain the consistency of the engraving depth, and the adjustment flow chart is shown in Figure 4.

During the engraving process, the real-time parameters such as scanning velocity vector, surface normal, and laser beam attitude are obtained from the CNC system. The current local engraving coordinate system frame can be constructed according to the scanning velocity vector and surface normal. Then, the laser incident angle and scanning angle of the beam axis relative to the local frame are calculated. Under the target depth, the optimal laser power can be calculated based on the proposed engraving depth model. Finally, the corresponding PWM signal is generated and sent to the CO_2_ laser in real time to adjust the laser output power.

An variable-angle straight groove laser engraving experiment was designed to verify the proposed method of laser power adjustment. The trajectory of the straight groove was a straight plane line, and the laser incident angle and scanning angle changed with the position of the straight line, as shown in Equation (1).

(1) $$\left\{ \begin{array}{l} x=40u\\ y=0\\ \theta =30{}^{\circ}\times u\\ \varphi =60{}^{\circ}\times u \end{array}\right.,u\in \left[0,1\right]$$

where *x* is the X position of the trajectory curve, *y* is the Y position, θ is the laser incident angle, φ is the laser scanning angle, and *u* is the curve parameter.

**Figure 5:**
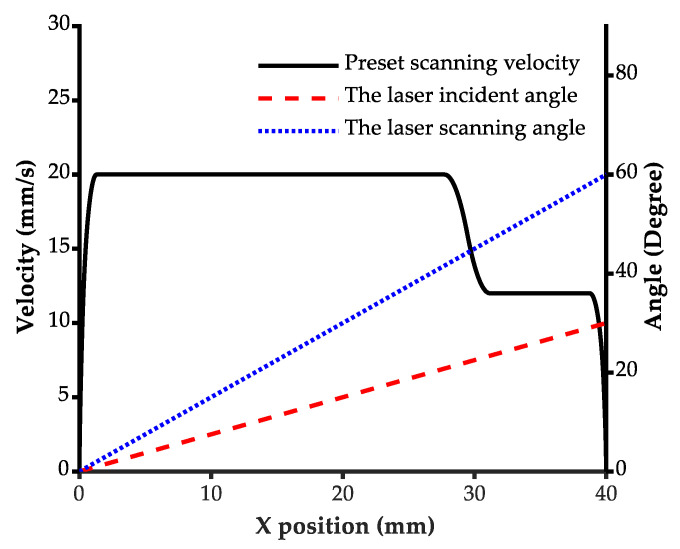
The preset scanning velocity and laser beam angles in the straight groove laser engraving experiment.

The preset scanning velocity curve was generated by the S-shape feed-rate plan method according to the constraints of the velocity command of 20 mm/s, the maximum acceleration of 1000 mm/s, and the maximum jerk of 5000 mm/s. In addition, the velocity command was reduced from 20 mm/s to 12 mm/s at u = 0.7 to simulate a significant decrease in the scanning velocity at the corner. The preset velocity curve and the beam angle are shown in Figure 5. The experiment was carried out on the experimental platform according to the preset scanning velocity curve, while the laser power command was adaptively controlled based on different depth models to maintain the normal depth at the target depth 310 μm.

## 3. Depth Model of 5-Axis Variable-Angle Laser Engraving 

### 3.1. Depth Model of 5-Axis Variable-Angle Laser Engraving Based on the Energy Conservation Principle

**Figure 6:**
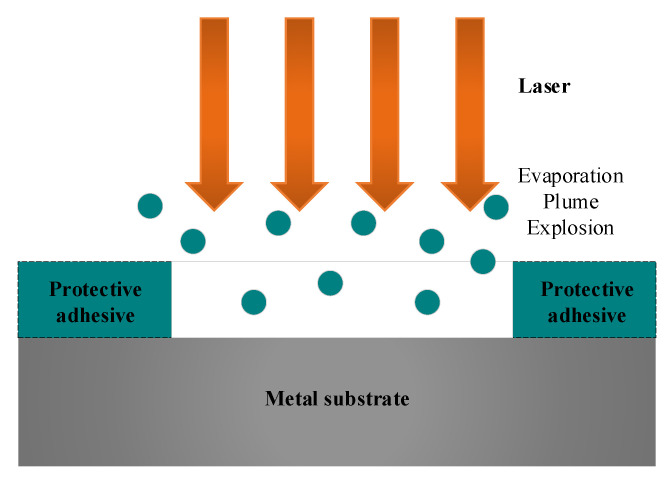
Mechanism diagram of laser engraving.

Laser engraving is a laser beam machining application based on laser ablation. The laser beam is irradiated on the part surface to remove the protective adhesive without damaging the substrate material. The primary mechanism of laser engraving is that the thermal effect generated by the laser beam removes the protective adhesive by evaporation, plume, and explosion [12], as shown in Figure 6.

According to the energy conservation principle, the laser energy absorbed by the adhesive surface in a period, $\Delta E_{L}$, equals the heat consumed in the laser engraving process, $\Delta Q_{c}$.

(2) $$\Delta E_{L}=\Delta Q_{c}$$

At the same time, the laser energy absorbed by the adhesive surface can be expressed as:

(3) $$\Delta E_{L}=AP_{L}\Delta t$$

where A is the absorption rate of the adhesive layer material to the laser beam, $P_{L}$ is the average output power of the laser beam, and Δt is the processing time.

The heat consumed in the laser engraving process is the sum of the heat absorbed by the material sublimation process and the heat lost by the heat conduction of the surrounding materials [30]:

(4) $$\Delta Q_{c}=\rho \Delta V\left[c_{p}\left(T_{V}-T_{\infty }\right)+H_{m}+H_{V}\right]+\Delta Q_{HL}$$

where ΔV is the removed volume of the protective adhesive material in time Δt, ρ is the density of adhesive, $c_{p}$ is the specific heat enthalpy, $T_{V}$ is the evaporation temperature, $T_{\infty }$ is the ambient temperature, $H_{m}$ is the specific melting enthalpy, $H_{V}$ is the specific evaporation enthalpy, and $\Delta Q_{HL}$ is the heat lost by the heat conduction of the surrounding materials.

The relationship between laser output power and the removed volume of the adhesive material in laser engraving can be obtained by combining Equations (2)–(4) and taking the limit of the equation divided by time Δt.

(5) $$AP_{L}=\frac{dV}{dt}\rho \left[c_{p}\left(T_{V}-T_{\infty }\right)+H_{m}+H_{V}\right]+\frac{dQ_{HL}}{dt}$$

The adhesive material is removed as the laser beam scans along the trajectory; therefore, the removed volume, *V*, can be calculated by integrating the normal cross-sectional area along the scanning trajectory:

(6) $$V=\int Sv_{c}dt$$

where S the normal section area of the engraved line and $v_{c}$ is the scanning velocity of the laser focus relative to the surface.

According to the Newton–Leibniz formula, the relationship in Equation (5) can be expressed as:

(7) $$AP_{L}=Sv_{c}\rho \left[c_{p}\left(T_{V}-T_{\infty }\right)+H_{m}+H_{V}\right]+\frac{dQ_{HL}}{dt}$$

Assuming the laser beam is the intensity distribution of the Laguerre–Gaussian mode, $TEM_{0}^{0}$, and the polarization state is circular polarization, the light intensity distribution of the focus spot is as follows:

(8) $$I\left(r\right)=I_{m}e^{-\frac{r^{2}}{2\sigma^{2}}}$$

where I(r) is the light intensity at the position with distance *r* from the beam axis, $I_{m}$ is the maximum light intensity, and σ is the standard deviation of the light intensity under Gaussian distribution.

The spot radius $r_{f}$ is generally estimated according to the distance to the beam axis when the light intensity is reduced to $\frac{1}{e^{2}}$ of $I_{m}$. It can be obtained as:

(9) $$r_{f}=2\sigma$$

**Figure 7:**
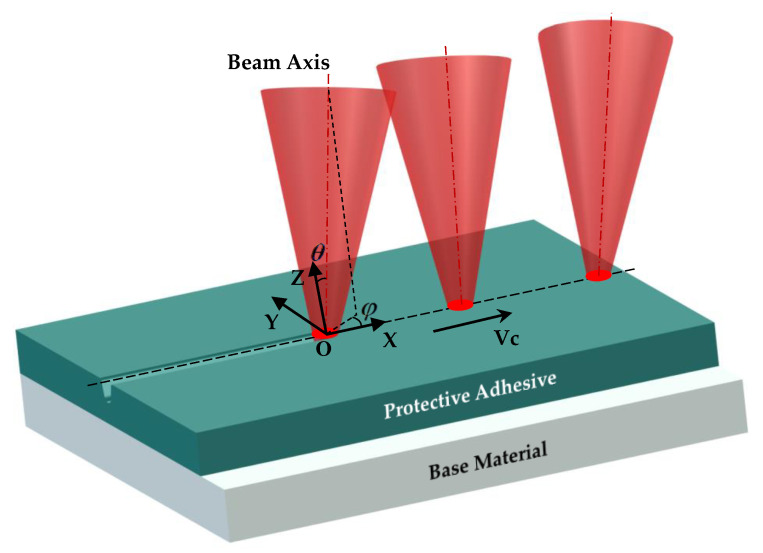
The schematic diagram of 5-axis variable-angle laser engraving.

The laser engraving trajectories of the chemically milled parts with complex structural characteristics are usually complex curves, and the angle of the laser beam axis changes continuously during the laser processing. The local coordinate frame OXYZ is constructed using the scanning velocity direction vector and the surface normal vector, as shown in Figure 7. The laser incident angle is the angle between the laser beam axis and the Z axis, denoted by θ. The laser scanning angle is the angle between the laser incidence plane and the scanning velocity direction vector, denoted by φ.

**Figure 8:**
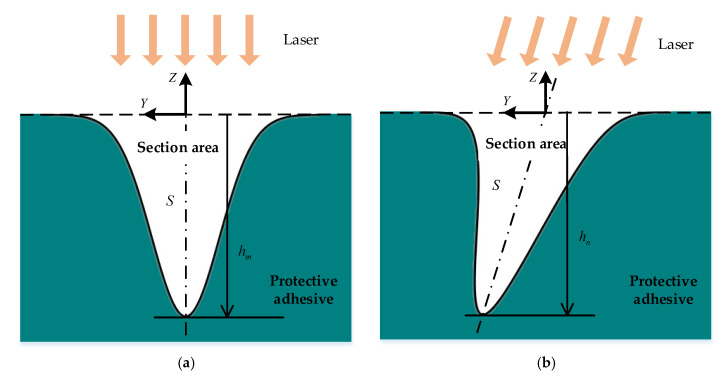
Section profile shapes of 5-axis variable-angle laser engraving. (**a**) Laser scanning normally to the surface; (**b**) laser scanning obliquely to the surface.

The energy of the laser accumulated in the scanning section during the scanning process follows the same Gaussian distribution as when the laser beam scans in the normal direction. Then, the depth of the scribed section also follows the same Gaussian distribution according to the principle of energy conservation, as shown in Figure 8a. Set the maximum depth of the section as $h_{m}$, the area of the engraved line normal section can be calculated as follows:

(10) $$S=\int_{-\infty }^{+\infty }h\left(x\right)dx=\sqrt{2\pi }\sigma h_{m}=\sqrt{\frac{\pi }{2}}r_{f}h_{m}$$

The spot shape of the laser beam irradiated on the adhesive surface is an ellipse during the 5-axis variable-angle laser engraving when both the incident angle and the scanning angle are not zero, as shown in Figure 8b. Here, assume that the profile shape axis of the normal section is inclined to the surface normal. The area of the normal section, in this case, can be approximately calculated by:

(11) $$S=\sqrt{\frac{\pi }{2}}r_{s}h_{n}$$

where $r_{s}=r_{f}\sqrt{\cos^{2}\varphi +\sec^{2}\theta \sin^{2}\varphi }$ is the half-width of the track formed by the elliptical spot scanning, which can be calculated according to the geometric relationship of the conic section. $h_{n}$ is the maximum depth of the engraved line in the normal direction of the surface, which is a critical parameter to evaluate the laser engraving result. The normal cross-sectional area of the 5-axis variable-angle laser engraving can be expressed as:

(12) $$S=\sqrt{\frac{\pi }{2}}r_{f}h_{n}\sqrt{\cos^{2}\varphi +\sec^{2}\theta \sin^{2}\varphi }$$

Combining Equations (7) and (12), the energy balance equation in the process of 5-axis variable-angle laser engraving can be written as:

(13) $$AP_{L}=\sqrt{\frac{\pi }{2}}\rho \left[c_{p}\left(T_{V}-T_{\infty }\right)+H_{m}+H_{V}\right]r_{f}h_{n}v_{c}\sqrt{\cos^{2}\varphi +\sec^{2}\theta \sin^{2}\varphi }+\frac{dQ_{HL}}{dt}$$

The material characteristic parameters such as ρ, $c_{p}$, $T_{V}$, $T_{\infty }$, $H_{m}$, $H_{V}$, and the laser beam parameter, $r_{f}$, are constant during the laser engraving process under the same processing conditions of the material and the environment. The above parameters can be replaced by a constant. The heat conduction loss, $Q_{HL}$, is dependent on the cutting parameters [31]. Setting the exponent of the scanning velocity in the energy balance equation to a variable parameter, α, Equation (13) can be simplified to:

(14) $$AP_{L}=\eta h_{n}v_{c}{}^{\alpha }\sqrt{\cos^{2}\varphi +\sec^{2}\theta \sin^{2}\varphi }+P_{res}$$

where η is a constant and $P_{res}$ is the residual rate of thermal conduction loss after model simplification. 

The laser absorptivity of the material usually changes with the laser incident angle, which can be approximated by a quadratic polynomial of the incident angle, θ. Dividing both sides of Equation (14) by parameter η, the 5-axis variable-angle laser engraving depth model can be obtained as:

(15) $$\left(\xi_{0}+\xi_{1}\theta +\xi_{2}\theta^{2}\right)P_{L}=h_{n}v_{c}{}^{\alpha }\sqrt{\cos^{2}\varphi +\sec^{2}\theta \sin^{2}\varphi }+C_{res}$$

where $\xi_{0}$, $\xi_{1}$, $\xi_{2}$, and $C_{res}$ are the coefficients of the depth model, which are determined by the material characteristics, processing conditions, and laser beam parameters. The depth model parameters can be identified by the experimental data of laser engraving. 

### 3.2. Identification of the Depth Model Parameters

When the laser incident angle is 0, the depth model in Equation (15) can be written as follows.

(16) $$\xi_{0}P_{L}-C_{res}=h_{n}v_{c}^{\alpha }$$

Taking the logarithm of both sides of Equation (16):

(17) $$\ln\left(P_{L}-\frac{C_{res}}{\xi_{0}}\right)-\ln h_{n}=\alpha \ln v_{c}-\ln\xi_{0}$$

The optimal solution of the parameters α and $-\ln\xi_{0}$ can be estimated as follows by the least square method:

(18) $$\left[\begin{array}{c} \alpha \\ -\ln\xi_{0} \end{array}\right]={\left(X_{\alpha }^{T}X_{\alpha }\right)}^{-1}X_{\alpha }^{T}y_{\alpha }$$

where $X_{\alpha }$ is the coefficient matrix and $y_{\alpha }$ is the value vector. Both are obtained by the test data when the laser incident angle is 0 as follows:

(19) $$X_{\alpha }=\left[\begin{array}{cc} \ln v_{c,1} & 1\\ \ln v_{c,i} & 1\\ \vdots & \vdots \\ \ln v_{c,m} & 1 \end{array}\right],y_{\alpha }=\left[\begin{array}{c} \ln\left(P_{L,1}-C_{res}/\xi_{0}\right)-\ln h_{n,1}\\ \ln\left(P_{L,i}-C_{res}/\xi_{0}\right)-\ln h_{n,i}\\ \vdots \\ \ln\left(P_{L,m}-C_{res}/\xi_{0}\right)-\ln h_{n,m} \end{array}\right]$$

In the same way, the parameters $\left(\xi_{0},\xi_{1},\xi_{2},C_{res}\right)$ of the proposed depth model can be estimated after determining the parameter α.

(20) $${\left[\begin{array}{cccc} \xi_{0} & \xi_{1} & \xi_{2} & C_{res} \end{array}\right]}^{T}={\left(X^{T}X\right)}^{-1}X^{T}y$$

where *X* is the coefficient matrix and *y* is the value vector. Both are obtained by the test data as follows.

(21) $$X=\left[\begin{array}{cccc} P_{L,1} & \theta_{1}P_{L,1} & \theta_{1}^{2}P_{L,1} & -1\\ P_{L,i} & \theta_{i}P_{L,i} & \theta_{i}^{2}P_{L,i} & -1\\ \vdots & \vdots & \vdots & \vdots \\ P_{L,n} & \theta_{n}P_{L,n} & \theta_{n}^{2}P_{L,n} & -1 \end{array}\right],y=\left[\begin{array}{c} h_{n,1}v_{c,1}^{\alpha }\sqrt{\cos^{2}\varphi_{1}+\sec^{2}\theta_{1}\sin^{2}\varphi_{1}}\\ h_{n,i}v_{c,i}^{\alpha }\sqrt{\cos^{2}\varphi_{i}+\sec^{2}\theta_{i}\sin^{2}\varphi_{i}}\\ \vdots \\ h_{n,n}v_{c,n}^{\alpha }\sqrt{\cos^{2}\varphi_{n}+\sec^{2}\theta_{n}\sin^{2}\varphi_{n}} \end{array}\right]$$

$C_{res}$ is set to 0 when estimating parameter α by Equation (18) for the first time. After obtaining the parameters $\left(\xi_{0},\xi_{1},\xi_{2},C_{res}\right)$ by Equation (20), $C_{res}$ and $\xi_{0}$ must be substituted into Equation (18) to re-estimate parameter α to reduce the identification error. The above process is iterated until the difference between the identified values of the parameter α meets the following condition.

(22) $$\left|\alpha_{k}-\alpha_{k-1}\right|<\delta_{\alpha }$$

## 4. Results and Discussions

### 4.1. Results of the Orthogonal Test

The normal depths of the engraved lines of the orthogonal test were calculated by the statistical method and are listed in Table 5.

The parameters of the 5-axis variable-angle laser engraving depth were are identified by the least square method using the experimental data. Table 6 shows these results.

The laser engraving depth model using the laser engraving experimental platform under current experimental conditions is expressed as follows.

(23) $$h_{n}=\frac{\left(812+169.7\theta -384.2\theta^{2}\right)P_{L}+1171}{v_{c}^{0.8477}\sqrt{\cos^{2}\varphi +\sec^{2}\theta \sin^{2}\varphi }}$$

where the unit of $h_{n}$ is micrometer, the unit of $v_{c}$ is millimeter per second, the unit of $P_{L}$ is watt, and the units of θ and φ are radian.

**Figure 9:**
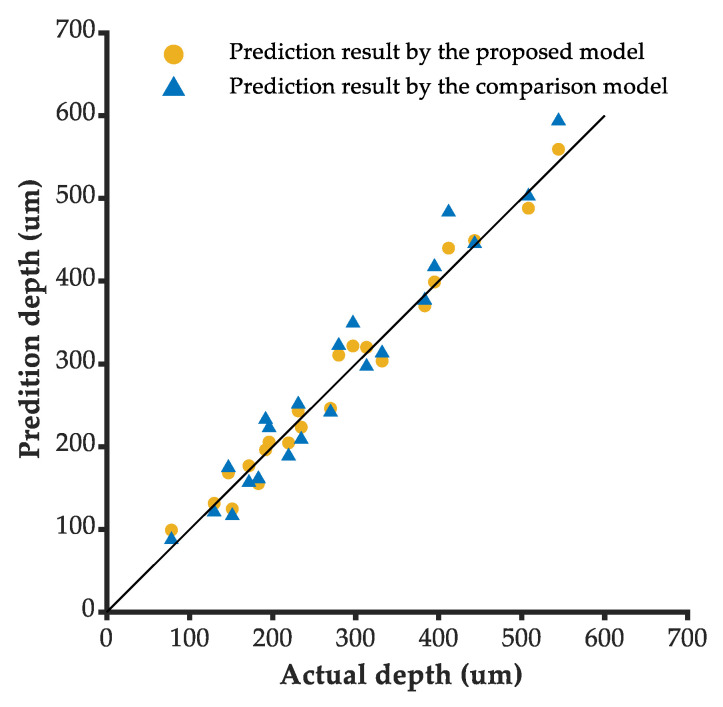
Distributions of the prediction results relative to actual values.

The engraving depths of the orthogonal test parameters in Table 3 are predicted based on the proposed depth model and the comparison model [13]. It can be seen from Figure 9 that the distribution of the predicted results by the proposed model relative to actual values is closer to the symmetric line than that of the prediction results by the comparison model.

The root mean square error (RMSE) can be used to evaluate the prediction accuracy of the model, which is expressed as follows:

(24) $$RMSE=\sqrt{\frac{1}{n}\sum_{i=1}^{n}{\left(h_{i}-{\hat{h}}_{i}\right)}^{2}}$$

where $h_{i}$ is the actual normal depth of the *i*th test, ${\hat{h}}_{i}$ is the normal depth of model prediction by the *i*th test parameters, and *n* is the number of tests. 

The coefficient of determination, $r^{2}$, is a dimensionless statistical index to reflect the reliability of the model to describe the relationship between the dependent variable and independent variables, which is expressed as follows:

(25) $$r^{2}=1-\frac{\sum_{i=1}^{n}{\left(h_{i}-{\hat{h}}_{i}\right)}^{2}}{\sum_{i=1}^{n}{\left(h_{i}-\bar{h}\right)}^{2}}$$

where $\bar{h}$ is the average actual normal depth of the tests.

The prediction root mean square error of the proposed model is 18.6 μm, while that of the comparative model is 31.2 μm, as shown in Table 7. The prediction accuracy of the proposed depth model is better in the 5-axis laser engraving process. The coefficient of determination of the proposed model is 0.977; closer to 1 than that of the comparative model, which has a value of 0.936. The proposed depth model better describes the relationship between the engraving depth and the processing parameters.

### 4.2. Laser Adjustment Results of the Variable-Angle Straight Groove Engraving

**Figure 10:**
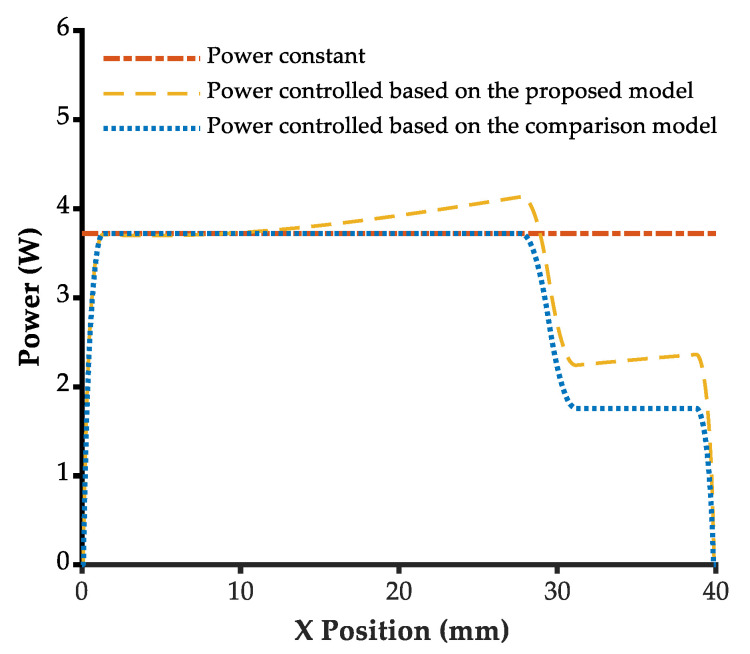
The laser power results controlled by different methods.

The laser power in the variable-angle straight groove engraving experiment was adjusted based on constant power, the proposed model, and the comparison model, respectively, to maintain the normal depth at the target depth of 310 μm, as shown in Figure 10. The constant power command was set to 3.72 W throughout the engraving process. The power command controlled based on the comparison model changed with the scanning velocity, while the power command controlled based on the proposed model was larger because of the beam angle variation.

**Figure 11:**
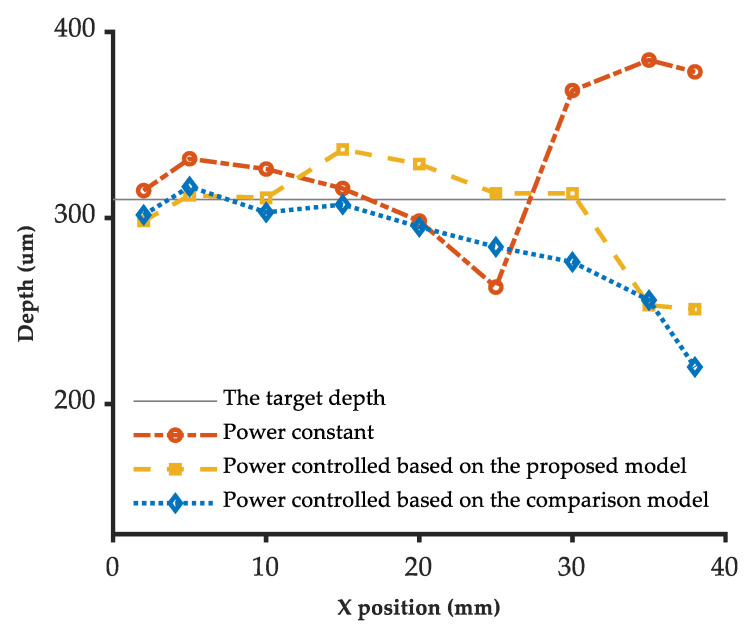
The normal depth results of the variable-angle straight groove.

The ultra-depth microscope measured the 3D profiles at nine selected locations on the engraved line. Table 8 shows the engraving depths results under different laser power control methods. It can be seen from Figure 11 that the depth of constant power engraving decreases gradually in the first uniform velocity scanning period and increases significantly after the deceleration of the scanning velocity. The engraving depth controlled based on the proposed model fluctuates near the target depth (310 μm), and the engraving depth by the power controlled based on the comparison model gradually decreases from about 310 μm to about 220 μm during the whole process.

The average depth of the straight groove was estimated statistically using the normal depths in Table 8. The relative error compared with the target depth is calculated by Equation (26):

(26) $$\delta =\frac{{\bar{h}}_{n}-h_{n0}}{h_{n0}}\times 100\%$$

where ${\bar{h}}_{n}$ is the overall average normal depth of the straight groove and $h_{n0}$ is the target normal depth. 

As shown in Table 9, the results indicate that the relative error compared with the target depth based on the proposed model (−2.6%) is the lowest among the three methods. The standard deviation of the proposed model (30.4 μm) is the same as that of the comparison model and better than that of the constant power experiment (40.0 μm). Therefore, the laser power adaptive control method based on the 5-axis variable-angle laser engraving depth model can effectively keep the engraving depth near the target depth in the case of sharp changes in the laser beam axis angle and scanning velocity.

## 5. Conclusions

A depth model of 5-axis variable-angle laser engraving is established based on the principle of energy conservation. It clarifies the relationship between the laser beam axis angle, scanning velocity, laser power, and the engraving depth, which supports the real-time adaptive adjustment of laser power with scanning velocity and beam axis angles under the target depth constraint. In order to verify the validity of the proposed model and method, an orthogonal experiment and a 5-axis variable-angle straight groove engraving experiment were carried out. The experimental results show that the 5-axis variable-angle laser engraving depth model established in this paper is superior to the comparison model in both the simulation prediction accuracy and the straight groove engraving quality. The proposed depth model is carried out on the premise that the laser beam is circularly polarized and the intensity distribution is the Laguerre–Gaussian mode. Further work can consider the influence of a more general laser beam on the engraving depth. The engraving width model can also be studied, which helps evaluate and improve the contour accuracy of pattern engraving.

## Figures and Tables

**Table 1:** The research status of engraving depth models.

Method	Scholars	Research Content
The engraving depth model based on the laser ablation mechanism	Arnold, N et al. [13]	Established the relationship between laser ablation depth and laser energy density of polymer materials based on the photothermal melting zone fracture theory.
	Prakash, S. et al. [14], Shahbazi et al. [15], Zhao, K. et al. [16]	Studied the correlation among multi-pulse laser ablation depth, laser energy, and feed-rate through the superposition of multi-pulse laser ablation depth based on the principle of energy balance.
	Nakamura S et al. [17], Arkadiusz A.J [18], Pazokian H [19]	Established the ablation theoretical model of tetrafluoroethylene, hexafluoropropylene, nylon, and polymers, and studied the relationship between laser beam intensity, pulse repetition rate, and material scanning velocity.
The engraving depth model based on regression analysis	Bovatsek J.M et al. [20], Jia, Z et al. [21], Xiaowei B. et al. [22]	Studied the relationship between depth, width, processing quality, and machining parameters by controlling variate methods and the linear regression method.
	Ai J et al. [23]	Systematically studied the influence of laser incident angle on the size accuracy of laser processing patterns and the influence of scanning times on the defocused laser processing line width by the orthogonal experimental method and linear regression method.
	Desai, C.K. et al. [24]	Established an etching depth model in thermoplastic micro-milling by non-linear regression method by considering material properties, laser power, and cutting velocity.
The engraving depth prediction model based on the artificial intelligence method	Nukman, Y. et al. [25], Yin, Z. et al. [26], Smokvina H.S. et al. [27]	Established the laser engraving depth models through artificial neural networks and other artificial intelligence methods.
	Hossain, A. et al. [28], Juez-Gil et al. [29]	Built-up an intelligent fuzzy expert system (FES) model and multilayer-perceptron hybrid strategy to predict the kerf width in CO2 laser cutting.

**Table 2:** Parameters of the laser beam.

Parameter	Value
Wavelength	10.6 μm
Output Power	0–30 W
Operating Frequency	0–20 KHz
Power Stability	±5%
Beam Quality M^2^	<1.2
Focus Diameter	150 μm
Focus Depth	583 μm

**Table 3:** Actual average power at different duty cycles.

**Duty Cycle (%)**	3	5	7	9	11	13	15
**Average Power (W)**	1.55	2.31	3.08	3.72	4.53	5.11	5.79
**Standard Deviation (W)**	0.12	0.12	0.11	0.11	0.13	0.17	0.11

**Note:** Each effective measurement time is 15s. The pulse repetition frequency of the PWM signal is set to 2000 Hz.

**Table 4:** Orthogonal test parameters for laser engraving (the laser pulse frequency is 2000 Hz).

Test No.	Duty Cycle (%)	Scanning Velocity (mm/s)	Incident Angle (deg.)	Scanning Angle (deg.)	Test No.	Duty Cycle (%)	Scanning Velocity (mm/s)	Incident Angle (deg.)	Scanning Angle (deg.)
1	3	10	0	90	12	6	30	32	45
2	6	15	8	90	13	9	40	0	45
3	9	20	16	90	14	6	40	24	30
4	12	30	24	90	15	12	15	0	30
5	15	40	32	90	16	15	20	8	30
6	9	30	8	60	17	3	30	16	30
7	12	40	16	60	18	12	20	32	0
8	3	15	32	60	19	15	30	0	0
9	6	20	0	60	20	3	40	8	0
10	15	15	16	45	21	6	10	16	0
11	3	20	24	45	22	9	15	24	0

**Table 5:** Depth results of the orthogonal test.

**Test Number**	1	2	3	4	5	6	7	8
**Depth (W)**	297	280	332	231	196	234	219	191
**Standard** **Deviation (W)**	10.1	15.1	11.4	9.69	10.6	13.2	9.73	11.8
**Test Number**	9	10	11	12	13	14	15	16
**Depth (W)**	270	544	147	183	171	129	508	443
**Standard** **Deviation** **(W)**	15.8	10.2	13.5	9.52	8.68	11.5	22.2	9.12
**Test Number**	17	18	19	20	21	22		
**Depth (W)**	151	383	313	78	412	395		
**Standard** **Deviation (W)**	11.1	11.1	13.0	13.0	21.2	13.5		

**Table 6:** Parameter identification results of 5-axis variable-angle laser engraving depth model (all parameters are dimensionless).

**Parameter**	α	$\xi_{0}$	$\xi_{1}$	$\xi_{2}$	$C_{res}$
**Value**	0.8477	812.0	169.7	−384.2	−1171

**Table 7:** The evaluation results of the model predictions.

**Model**	The proposed model	The comparison model
**RMSE (μm )**	18.6	31.2
$r^{2}$	0.977	0.936

**Table 8:** Depth results of the variable-angle straight groove engraving.

**Measurement Number**		1	2	3	4	5	6	7	8	9
**X Position (mm)**		2	5	10	15	20	25	30	35	38
**Constant Power**	Depth (μm )	315	332	326	316	298	263	369	385	379
	Standard Deviation (μm )	25.4	26.3	18.3	15.4	17.9	22.1	32.9	15.4	12.1
**Power controlled based on the proposed model**	Depth (μm )	298	312	311	337	329	313	313	253	251
	Standard Deviation (μm )	25.2	14.4	11.1	12.4	19.1	22.2	33.1	15.5	9.74
**Power controlled based on the comparison model**	Depth (μm )	302	317	303	307	295	285	276	256	220
	Standard Deviation (μm )	28.7	25.4	12.8	13.6	18.4	19.4	25.2	23.2	17.5

**Table 9:** The average depth results of the 5-axis variable-angle straight groove engraving.

Power Type	Average Depth (μm )	Standard Deviation (μm )	Relative Error δ (%)
Constant Power	331.3	40.0	6.9
Power controlled by the proposed model	302.0	30.4	−2.6
Power controlled by the comparison model	284.5	30.4	−8.2

## Data Availability

Not applicable.
